# Evolutionary conservation of regulated longevity assurance mechanisms

**DOI:** 10.1186/gb-2007-8-7-r132

**Published:** 2007-07-05

**Authors:** Joshua J McElwee, Eugene Schuster, Eric Blanc, Matthew D Piper, James H Thomas, Dhaval S Patel, Colin Selman, Dominic J Withers, Janet M Thornton, Linda Partridge, David Gems

**Affiliations:** 1 Department of Biology, University College London, London WC1E 6BT, UK; 2 Department of Genome Sciences, University of Washington, Seattle, Washington 98195-5065, USA; 3 European Bioinformatics Institute, Hinxton CB10 1SD, UK; 4 Department of Medicine, University College London, London WC1E 6BT, UK

## Abstract

**Background:**

To what extent are the determinants of aging in animal species universal? Insulin/insulin-like growth factor (IGF)-1 signaling (IIS) is an evolutionarily conserved (public) regulator of longevity; yet it remains unclear whether the genes and biochemical processes through which IIS acts on aging are public or private (that is, lineage specific). To address this, we have applied a novel, multi-level cross-species comparative analysis to compare gene expression changes accompanying increased longevity in mutant nematodes, fruitflies and mice with reduced IIS.

**Results:**

Surprisingly, there is little evolutionary conservation at the level of individual, orthologous genes or paralogous genes under IIS regulation. However, a number of gene categories are significantly enriched for genes whose expression changes in long-lived animals of all three species. Down-regulated categories include protein biosynthesis-associated genes. Up-regulated categories include sugar catabolism, energy generation, glutathione-S-transferases (GSTs) and several other categories linked to cellular detoxification (that is, phase 1 and phase 2 metabolism of xenobiotic and endobiotic toxins). Protein biosynthesis and GST activity have recently been linked to aging and longevity assurance, respectively.

**Conclusion:**

These processes represent candidate, regulated mechanisms of longevity-control that are conserved across animal species. The longevity assurance mechanisms via which IIS acts appear to be lineage-specific at the gene level (private), but conserved at the process level (or semi-public). In the case of GSTs, and cellular detoxification generally, this suggests that the mechanisms of aging against which longevity assurance mechanisms act are, to some extent, lineage specific.

## Abstract 2

Short abstract: A multi-level cross-species comparative analysis of gene-expression changes accompanying increased longevity in mutant nematodes, fruit flies and mice with reduced insulin/IGF-1 signaling revealed candidate conserved mechanisms.

## Background

Growth and development in living organisms, from bacteria to higher animals, are genetically programmed processes involving molecular mechanisms, many of which are evolutionarily ancient and shared across a broad range of taxa. Consequently, it is possible to understand genes and processes controlling mammalian growth and development by studying invertebrate model organisms such as the nematode *Caenorhabditis elegans *and the fruitfly *Drosophila melanogaster*. This is also true of other functions, such as cellular metabolism and neurobiology. But what about aging? According to evolutionary theory, aging is not a genetically programmed process, but rather a side-effect either of mutation pressure [1] or of selection for early life traits that enhance fitness [2]. From this, it is not clear that aging in different taxa will involve similar mechanisms [3]. Gross pathologies of aging certainly can differ greatly in different organisms: humans can die from stroke and cancer, while nematodes and fruit flies do not. There are at least some differences at the molecular level too: for example, accumulation of extrachromosomal ribosomal DNA circles contribute to aging in budding yeast (*Saccharomyces cerevisiae*) [4], and extrachromosomal mitochondrial DNA circles (senDNAs) to aging in the filamentous fungus *Podospora anserina *[5]; neither contribute to aging in mammals. Thus, at least some mechanisms of aging are private (lineage-specific) rather than public (evolutionarily conserved) [6].

However, recent studies have shown that the insulin/insulin-like growth factor (IGF)-1 signaling (IIS) pathway is a public determinant of aging. For example, mutation of the insulin/IGF-1 receptor *daf-2 *in *C. elegans *(GenBank: NM_065249), the insulin/IGF-1 receptor *dINR *and insulin-receptor substrate (IRS) *chico *in *Drosophila *(GenBank: NM_164899), and the IGF-1 and insulin receptors in mice can all increase lifespan [7-12]. Additionally, mutations in mice that decrease levels of circulating insulin and IGF-1, such as Prop-1^df/df ^and Ghrhr^lit/lit ^(the Ames and Little dwarf mice), also increase lifespan [13,14].

It has been demonstrated in *C. elegans *that IIS exerts effects on longevity via regulated effector genes [15-18]. That regulation of longevity by IIS is public could imply that such effectors are also public. Alternatively, IIS could control lifespan through mechanisms that differ between lineages. Resolving these possibilities is important, both for understanding the biological processes that can determine lifespan and for identifying the contexts in which the use of animal models for studying human aging is appropriate.

To begin to address these questions, we have compared the genes that are transcriptionally regulated during IIS-linked lifespan extension in three animal species: *C. elegans*, *Drosophila *and the mouse, surveyed using oligonucleotide microarray analysis (Affymetrix). To do this we used a novel analytical approach to examine conservation of regulation in which conservation was viewed at each of three different levels: that of gene orthologs, that of paralogous gene sets, and that of broader gene classes (defined by InterPro or Gene Ontology (GO) categories). We find that, in contrast to the public role in aging of IIS itself, IIS-regulated genes are not conserved at the level of gene orthology or of paralogous gene groups. However, if IIS-regulated genes are compared across species at the level of gene category (in some cases, at a process level), cross-species similarities are visible. Notably, we see down-regulation of categories linked to protein synthesis, consistent with recent findings that lowered protein translation increases lifespan in the yeast *S. cerevisiae *[19] and *C. elegans *[20-22]. We also see up-regulation of broad spectrum cellular detoxification (that is, the phase 1, phase 2 xenobiotic or drug detoxification system), particularly the glutathione-S-transferases (GSTs). Links between this complex somatic maintenance system and longevity assurance have previously been seen, for example, in *C. elegans *[23,24]. In the case of cellular detoxification, a conserved role in longevity only at the process level is consistent with the fact that the genes involved are largely the products of lineage-specific expansion, such that orthology is non-existent. This suggests some degree of lineage specificity in the targets of detoxification, some of which may contribute to aging.

## Results

### Cross-species comparison of transcript profiles in long-lived mutants with reduced insulin/IGF-1 signaling

To search for public, IIS-regulated determinants of longevity, we used previously published microarray data from long-lived mutant worms and mice with lowered IIS, and generated new microarray data for a long-lived IIS mutant in flies (see Table 1 for array data overview). For each species, raw data were analyzed using rigorous quality control procedures and the same statistical methods to maximize data comparability (see Materials and methods) [25].

**Table 1:** Details of transcript profile datasets compared in this study

Organism	Genotypes compared	Sex	Age at sampling	Number of arrays per genotype	Reference
*C. elegans*	daf-2 vs daf-16; daf-2*	Hermaphrodite	1 day^†^	10	[24]
*D. melanogaster*	*chico*^1^/+ *vs *+/+	Female	7 days	5	This study
*M. musculus*	Prop-1^df/df ^*vs *+/+	Male	3 months	3	[26]
*M. musculus*	Ghrhr^lit/lit ^*vs *+/+	Male	3 months	3	[26]

* Data from five comparisons using either *daf-2(m577) *or *daf-2(e1370) *were pooled, giving a total of ten comparisons. *daf-16 *allele used: *mgDf50*. All strains also contained the temperature-sensitive sterile mutation *glp-4(bn2)*. ^†^Days of adulthood.

In *C. elegans*, the increased lifespan of *daf-2 *mutants requires the downstream FOXO transcription factor DAF-16 (GenBank: NM_001026423) [9]. We reanalyzed mRNA profile data comparing long-lived *daf-2 *mutants and non-long-lived *daf-16; daf-2 *double mutants, effectively a comparison of DAF-16 ON and DAF-16 OFF [24]. This identified 953 differentially expressed genes (558 up-regulated, 395 down-regulated in *daf-2*, *q *< 0.1, here and below). Other transcript profiles of *C. elegans *IIS-regulated genes are available [15,16], which closely resemble the gene lists studied here [24]; these lists were generated using a different microarray platform (spotted DNA arrays), and we therefore chose not to include them in our analysis.

For *Drosophila*, we compared wild-type (Dahomey) and long-lived *chico*^1^/+ heterozygotes [8]. This identified 1,169 differentially expressed genes (893 up-regulated, 276 down-regulated in *chico*^1^/+). Initially, we also examined transcript profiles from homozygous *chico*^1 ^mutants, which are slightly longer lived than *chico*^1^/+. However, the proportion of genes showing differential expression was so high as to make data analysis impracticable (data not shown). This difficulty was likely due to the fact that homozygous *chico*^1 ^flies are sterile dwarfs, with different quantities of eggs and oocytes, and altered allometry of tissues and organs and, as a result, the mRNAs that they contain. By contrast, *chico*^1^/+ flies are fertile and normal sized. Thus, the present analysis was only possible thanks to the semi-dominant effect of *chico*^1 ^on aging but not on fertility and size.

Finally, for the mouse, we reanalyzed data comparing gene expression in the liver of long-lived Prop-1^df/df ^(Ames dwarf) and Ghrhr^lit/lit ^(Little) mutants to normal-lived controls [26]. Both mutants fail to secrete growth hormone, and have little circulating IGF-1. While comprehensive array datasets from these models are currently only available for the liver, the liver in mammals is a crucial insulin-sensitive tissue. Moreover, the comparable tissues in worms (the intestine) and flies (the fat body) have both been shown to be specific mediators of the longevity of IIS mutants [27,28]. In our analysis, 1,416 genes were differentially expressed in the Ames dwarf (761 up-regulated, 655 down-regulated in the mutant), and 1,042 in the Little mouse (575 up-regulated, 467 down-regulated in the mutant).

If IIS controls aging via regulated public mechanisms, we would expect to see similarities between transcriptional changes in long-lived mutants in each species. We initially reasoned that such similarities could occur on either of two levels. Firstly, IIS could regulate a set of orthologous genes in all species. Secondly, IIS could regulate genes contributing to similar biological processes in different species (for example, antioxidant defence) that result in increased longevity. This might or might not involve orthologous genes in the three species.

### Absence of evolutionary conservation in IIS regulation at the gene level

For gene-level (as opposed to process-level) analysis, we first identified orthologous pairs of genes between each species, and orthologous sets of genes between all three species (Additional data file 4). We then screened for ortholog pairs or sets (triplets) that showed significant (*q *< 0.1) changes in expression in each species, and in the same direction (up- or down-regulated given reduced IIS). Surprisingly, very few orthologous genes changed expression co-ordinately in different species, and the number of such genes differed little from that expected by chance alone. For example, only nine ortholog pairs were significantly up-regulated in the worm and fly datasets (approximately 14 would be expected by chance). However, four ortholog sets were up-regulated in the worm, fly and Little mouse, significantly more (*p *= 0.003) than expected by chance alone (Tables 2, 3, 4).

**Table 2:** Simulation of expected number of differentially expressed ortholog sets: ortholog overview statistics

Category	Total	Up/down
Unique Ames mouse genes	7,188	3,517/3,671
Unique Little mouse genes	7,157	3,442/3,715
Unique fly genes	10,395	4,951/5,444
Unique worm genes	12,414	5,799/6,615
		
Worm-fly orthologs	3,588	NA
Worm-Ames orthologs	2,469	NA
Fly-Ames orthologs	3,125	NA
Fly-Little orthologs	3,105	NA
Worm-Little orthologs	2,464	NA
Worm-fly-Little orthologs	2,152	NA
Worm-fly-Ames orthologs	2,323	NA
		
DE unique genes, worm	953	558/395
DE unique genes, fly	1,169	893/276
DE genes, Ames	1,416	761/655
DE genes, Little	1,042	575/467

The number of unique genes for each dataset shows the number of remaining probe sets in each analysis following removal of non-reporting probe sets, promiscuous and orphan probe sets, and multiple probe sets that report the same gene (in each case, the most significant probe set was retained). Total orthologs: number of ortholog pairs/sets with expression data in each of the relevant datasets. Differentially expressed (DE) unique genes: number of significantly differentially expressed (at *q *< 0.1) unique genes in each dataset.

**Table 3:** Simulation of expected number of differentially expressed ortholog sets: probability of the observed number of differentially expressed orthologs

Category (orthologous pairs or sets)	Expected DE orthologs	Observed DE orthologs	*p *value
Fly-Ames, up-regulated	27.7	23	0.85
Fly-Ames, down-regulated	7.4	5	0.86
Fly-worm, up-regulated	13.8	9	0.94
Fly-worm, down-regulated	3	0	1
Worm-Ames, up-regulated	11.4	9	0.81
Worm-Ames, down-regulated	6.9	5	0.83
Fly-Little, up-regulated	20.9	34	0.004
Fly-Little, down-regulated	5.2	3	0.9
Worm-Little, up-regulated	8.6	9	0.5
Worm-Little, down-regulated	5	1	0.99
Worm-fly-Ames, up-regulated	0.9	0	1
Worm-fly-Ames, down-regulated	0.5	0	1
Worm-fly-Little, up-regulated	0.6	4	0.003
Worm-fly-Little, down-regulated	0.2	0	1

The number of differentially expressed (DE) ortholog pairs/sets expected by chance and actually observed for each indicated comparison. In all cases, the orthologs were significantly differentially expressed in each microarray dataset (*q *< 0.1), and showed the same direction of change (either up- or down-regulated). The number of expected DE orthologs was determined by simulation *in silico*, and the probability of identifying at least the number of observed orthologs was calculated from the simulation and is represented by the *p *value (see Materials and methods for *p *value calculations).

**Table 4:** Gene-level conservation of IIS-regulated transcriptional responses, and effects of RNAi on lifespan in *C. elegans*

Gene ID	Gene description	Percentage of vector control	*p *value	Microarray fold change	*p *value
R13H8.1/*daf-16*	FOXO transcription factor, acts downstream of *daf-2*	43	<0.0001	-	-
C10G11.5/*pnk-1*	Pantothenate kinase	26	<0.0001	3.81	0
**T25G3.4**	**Glycerol-3-phosphate dehydrogenase**	101	0.64	1.96	0.004
**F57C2.5**	**Contains similarity to strictosidine synthase**	100	0.34	1.65	0.001
C41C4.7	Ortholog of the human cystinosin gene	100	0.17	1.63	0.0001
F19H8.1/*tps-2*	Trehalose-6-phosphate synthase	100	0.90	2.28	0.007
F56D1.6/*cex-1*	Calexcitin, involved in serotonin-mediated responses	91	0.37	2.11	0.004
**Y105C5B.28/*gln-3***	**Glutamine synthetase**	92	0.25	2.00	0.006
F55D10.1	Orthologous to mannosidase, α, class 2B, member 1	103	0.046	2.96	0.0007
H03A11.1	Ortholog of a protein expressed in hematopoietic cells	83	0.012	1.59	0.0009

This table shows the nine worm-fly orthologous genes that show increased expression in response to reduced IIS (fold change in expression in *daf-2 *relative to *daf-16*; *daf-2 *shown). In bold: genes also differentially expressed in the Little mouse; a paralog of *pnk-1 *is also up-regulated in the Little mouse (additional Table 2 in Additional data file 3). For simplicity, only the gene name for the worm ortholog of the gene pair is shown. Only ortholog pairs (or triplets) that showed the same direction of change were considered, and at the level of significance used (*q *< 0.1), only up-regulated ortholog pairs were identified. To test for a possible role in longevity, expression of each individual gene was knocked down in *C. elegans *using RNAi; lifespans were compared to those of animals treated with control vector RNAi and calculated as a percentage of vector control (full lifespan data are available in Additional data file 5). The *p *value is the result of the log rank test comparing experimental lifespans to vector control. RNAi of R13H8.1/*daf-16 *was used as a positive control, but is not a differentially expressed orthologous gene.

To further test whether the nine worm-fly ortholog gene pairs might be longevity determinants, we reduced expression of each gene in *C. elegans *using RNA-mediated interference (RNAi) in the long-lived, RNAi-hypersensitive strain *rrf-3(pk1426)*; *daf-2(m577) *(Table 4; Additional data file 5). As a positive control we performed RNAi using *daf-16 *which, as expected, resulted in a large decrease in lifespan (57%). Of the test genes, RNAi of only one, the pantothenate kinase *pnk-1*, significantly shortened lifespan. However, *pnk-1 *RNAi also did this in a normal-lived control strain (data not shown), and it also causes sterility, larval arrest, and embryonic lethality [29]. The reduced lifespan may therefore reflect a requirement for *pnk-1 *for overall viability rather than prevention of aging. Pantothenic acid is a component of coenzyme A, the acetylated form of which plays a key role in the citric acid cycle. Pantothenate kinase catalyzes the first step in coenzyme A synthesis. In conclusion, the transcriptional response to reduced IIS shows very little evolutionary conservation at the level of gene orthology.

The lack of conservation seen at the level of gene orthology was unexpected. It led us to wonder whether perhaps, in some cases, IIS-regulated functions might be performed in different species by paralogous genes rather than orthologous ones. To this end, we looked at expression of paralogous genes in long-lived worms, flies and mice in two ways. Firstly, we examined all sets of paralogs where there was either *n *≤ 2 or *n *≤ 3 paralogous genes present in the gene list for each individual species (see Materials and methods). We counted the number of paralog sets (pairs, triplets or quadruplets) where at least one gene was differentially expressed in each species, and in the same direction. Secondly, we examined all paralog sets, whatever their size, and counted the number of paralog sets where a substantial number of genes showed differential expression in the same direction (we used the arbitrary cut-off of >50%). In addition, we counted again the number of orthologs with altered expression in more than one species, using the same statistics (see Materials and methods). For each of these four levels of conservation (ortholog set, paralog sets of size *n *≤ 2, *n *≤ 3 or any size), we asked whether the number of ortholog or paralog sets identified were more than expected by chance alone. To this end we performed bootstrap analysis on paralogous groups, comparing the observed number of differentially expressed paralogous groups with the numbers obtained by drawing the lists of differentially expressed groups at random (see Materials and methods).

The results of this analysis are shown in additional Table 1 in Additional data file 3. As before, at the level of orthology, there was no conservation of IIS regulation. When this analysis was loosened to include small and then large paralog groups, for most comparisons, there was still no significant conservation of IIS regulation. However, one triplet comparison showed an over-representation of IIS-regulated genes in all paralog comparisons: there were up-regulated genes in worms, flies and Little mice in four paralog sets (*p *= 0.01) (additional Table 1 in Additional data file 3). Data for the individual four genes in each of the four models examined are shown in additional Table 2 in Additional data file 3. The four paralog sets identified two proteins that we previously identified as IIS regulated in worms and flies: pantothenate kinase and glycerol-3-phosphate dehydrogenase. The two other paralog sets were, firstly, fructose-biphosphate aldolase and, secondly, beta-glucosidase, lactase phlorizinhydrolase and related proteins. Thus three-quarters of IIS-regulated paralog sets are linked to sugar metabolism. In summary, our analysis of paralog sets supports the unexpected conclusion that there is little evolutionary conservation between *C. elegans*, *Drosophila *or mouse of IIS regulation at the gene level.

### Conservation of regulation by IIS at the process level

**Figure 1:**
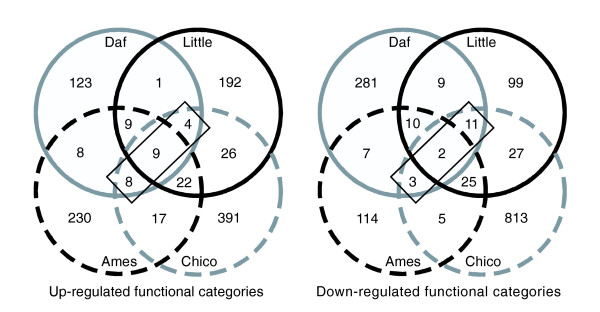
Overlap of differentially expressed functional categories in long-lived nematodes, fruitflies and mice. These Venn diagrams show the number and overlap of significantly differentially regulated functional categories (*p *< 0.05; GO categories and Interpro domain families) identified in each dataset using Catmap. While most of the differentially expressed categories in each dataset are species-specific, a small number of categories (boxed) show significant changes in expression in response to reduced IIS in all three species. These categories are detailed in Table 5.

Next we asked whether similar biochemical and cellular processes show conserved regulation at the transcriptional level by IIS. To this end we screened each dataset for biologically related genes or structurally related gene families showing co-ordinately increased or decreased expression in response to reduced IIS. Using biological annotation available through GO and Interpro, each dataset was analyzed using Catmap [30]. This software program assigns significance to gene categories based on their relative statistical ranking or representation within the dataset. This generated a list of gene categories showing significantly altered expression in each species; of these, a subset showed similar and significant changes in all three species (Figure 1; Table 5; Additional data file 6).

**Table 5:** Process-level conservation of IIS-regulated transcriptional responses

	Catmap *p *value			
	
	Worm	Fly	Mouse	
			
	*daf-2*	*chico*	Ames	Little
**Up-regulated Gene Ontology categories**				
GO:0008150 biological process				
GO:0046365 monosaccharide catabolism	***	**	*	NS
GO:0019320 hexose catabolism	***	**	*	NS
GO:0006007 glucose catabolism	***	**	*	NS
GO:0006090 pyruvate metabolism	*	*	**	*
GO:0006091 generation of precursor metabolites	*	***	***	***
GO:0015980 energy derivation by oxidation	***	**	*	**
GO:0006092 main pathways of carbohydrate metabolism	***	**	**	**
GO:0015849 organic acid transport	*	*	*	NS
GO:0046942 carboxylic acid transport	*	*	*	NS
GO:0005975 carbohydrate metabolism	**	***	**	***
GO:0044262 cellular carbohydrate metabolism	***	***	*	**
GO:0016052 carbohydrate catabolism	**	**	*	NS
GO:0044275 cellular carbohydrate catabolism	**	**	*	NS
GO:0003674 molecular function				
GO:0016491 oxidoreductase activity	***	***	***	***
GO:0016705 oxidoreductase activity with incorporation or reduction of molecular oxygen	*	**	NS	*
				
**Up-regulated Interpro categories**				
IPR000073 Alpha-beta hydrolase fold	*	*	NS	*
IPR001128 Cytochrome P450	***	***	*	NS
IPR002198 Short-chain dehydrogenase/reductase SDR	**	***	NS	**
IPR002347 Glucose-ribitol dehydrogenase	***	***	NS	***
IPR004045 Glutathione-S-transferase N-terminal	**	***	***	***
IPR004046 Glutathione-S-transferase C-terminal	**	***	***	***
				
**Down-regulated Gene Ontology categories**				
GO:0008150 biological process				
GO:0009059 macromolecular biosynthesis	*	***	**	*
GO:0006412 protein biosynthesis	**	***	***	**
GO:0043037 translation	*	***	*	NS
GO:0046907 intracellular transport	***	*	NS	*
GO:0006605 protein targeting	**	**	**	NS
GO:0006996 organelle organization and biogenesis	**	***	NS	*
GO:0007010 cytoskeleton organization/biogenesis	*	***	NS	*
GO:0007017 microtubule-based process	**	*	NS	*
GO:0009790 embryonic development	***	***	NS	*
GO:0043283 biopolymer metabolism	***	***	NS	*
GO:0003674 molecular function				
GO:0005488 binding	***	***	NS	*
GO:0003676 nucleic acid binding	*	***	NS	*
GO:0008135 translation factor, nucleic acid binding	*	***	NS	**
GO:0045182 translation regulator activity	*	***	NS	**
				
**Down-regulated Interpro categories**				
IPR000980 SH2 motif	***	***	*	NS
IPR002111 Cation not K+ channel TM region	*	*	NS	*

This table shows the functional categories that are significantly up- or down-regulated in response to reduced IIS in the worm, fly, and mouse (Ames and/or Little) microarray datasets. For brevity, the full hierarchy of the significant GO categories has not been shown. GO categories that fall directly under another significant category within the hierarchy are shown indented under the relevant category. Categories that fall into more than one hierarchy are only shown in one representative hierarchy. NS (non-significant; *p *> 0.05); *p < 0.05; **p < 0.005; ***p < 5.0e^-04^.

Next we tested whether the number of shared gene categories enriched for differentially regulated genes was more than predicted by chance alone. To do this, we performed bootstrap analysis of gene categories, drawing categories at random and computing *p *values from the number of common categories between the various combinations of gene lists (see Materials and methods). According to this analysis, for most comparisons the number of shared categories is more than predicted by chance alone, particularly where genes are up-regulated in the long-lived mutants (Additional data file 7). However, it should be borne in mind that the statistical test used assumes that the various categories are independent of one another, and in some cases this may not be the case. For example, cytochrome P450 (CYP) enzymes and GSTs can be subject to coordinate regulation [31]; moreover, given that the GO annotation is not a strict hierarchy, different GO categories may be non-independent. Thus, while the conclusion that no more gene classes are seen than expected by chance alone may be relied upon, the opposite conclusion cannot be. Nonetheless, the categories represented in Table 5 do potentially correspond to conserved IIS-regulated processes. These may include public determinants of aging that are not dependent on parallel transcriptional changes in orthologous genes.

An expected outcome of this analysis was that the two microarray datasets from the mouse would share more over-represented gene categories with one another than with the two invertebrate datasets. In terms of the individual genes showing altered expression, there are strong overlaps between the Prop-1^df/df ^and Ghrhr^lit/lit ^datasets [26]. However, the number of shared categories is surprisingly low (Figure 1). To some degree, this may reflect the fact that the Prop-1^df/df ^mutation is more pleiotropic, blocking production of thyroid stimulating hormone and prolactin in addition to growth hormone. It may also reflect the larger size of the lists of differentially expressed genes from the dwarf mouse studies, which can reduce the sensitivity of the test for overlapping gene categories. More positively, it suggests that comparing datasets from the two mouse strains has acted as a strong filter to exclude numerous gene categories unlinked to the increased lifespan phenotype.

**Figure 2:**
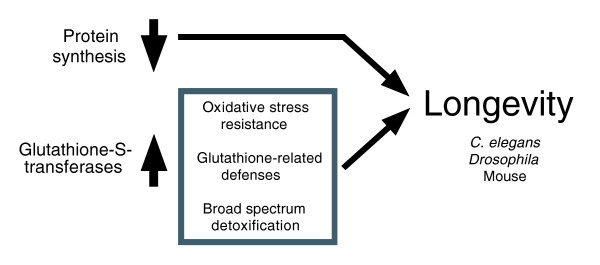
Protein synthesis and GST activity are potential semi-public determinants of longevity.

The majority of the common up-regulated GO categories are involved in sugar catabolism and energy generation (Table 5), implying that these processes are activated in IIS mutant animals. This is likely to reflect insulin-like control of sugar homeostasis by IIS in the three organisms. It is also consistent with a recent study of genes linked to energy metabolism in the worm dataset, which implies increased conversion of fat to carbohydrate and conservation of ATP stocks [32]. Among the shared down-regulated GO categories are many linked to protein biosynthesis and translation (Table 5), implying down-regulation of these processes in long lived milieus. Interestingly, it was recently discovered that lifespan in *C. elegans *is increased by loss of function of several genes promoting protein translation, including translation initiation factors and ribosomal proteins [20-22]. Thus, our results suggest that reduced protein translation may be a public mechanism of longevity assurance regulated by IIS (Figure 2).

**Figure 3:**
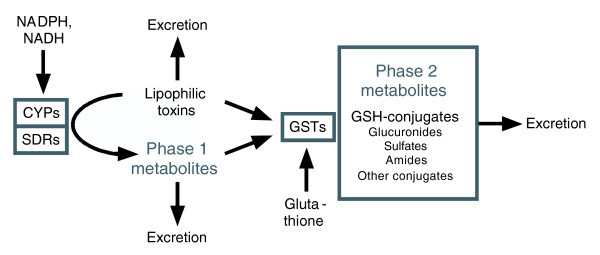
Cellular detoxification (drug metabolism). This process entails two phases: phase 1 (functionalization reactions), and phase 2 (conjugative reactions), which are carried out by several large and diverse gene families, including the CYPs, SDRs and GSTs.

Most of the Interpro domain gene families showing conserved up-regulation in IIS mutants are linked to cellular detoxification (that is, drug or xenobiotic metabolism) (Table 5; Figure 3). These correspond mainly to CYP, short-chain dehydrogenase/reductase (SDR; note that glucose-ribitol dehydrogenases are a type of SDR), and GST enzymes. Our analysis suggests the possibility that this detoxification system is a public mechanism of longevity assurance, protecting against the stochastic molecular damage that underlies the aging process.

### Random distribution of IIS-regulated genes among lineage-specific expansions of detoxification genes

**Figure 4:**
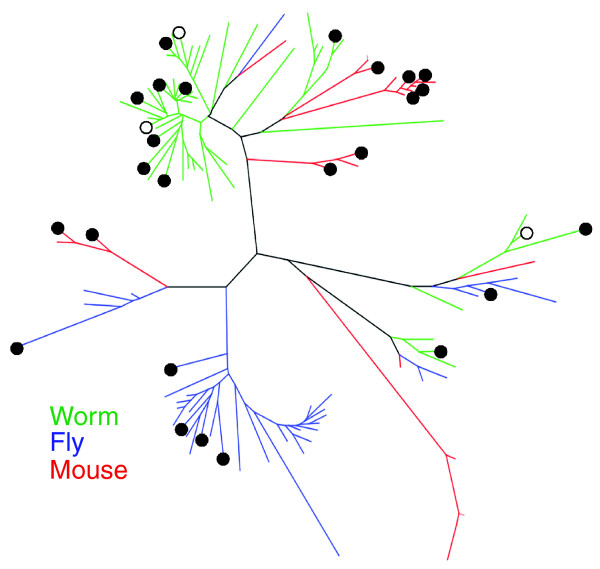
Phylogenetic tree of the GST gene families from worms, flies, and mice. Genes from each species are color-coded, and significantly (*q *< 0.1) differentially expressed genes in each dataset are shown by closed (up-regulated) or open (down-regulated) circles (see Additional data file 2 for phylogenetic trees for GST, CYP, SDR, and UGT gene families).

The association of increased expression of gene classes linked to cellular detoxification with longevity in three species, coupled with the lack of gene-level orthology, prompted us to examine the evolutionary relationships of these gene families in more detail. To do this, we constructed phylogenetic trees for each of three families in worms, flies, and mice, and then examined the distribution of IIS-regulated gene expression. Figure 4 shows a phylogenetic tree of worm, fly and mouse GSTs, marked to show differentially expressed genes (see also Additional data file 2). We also examined the phylogenetic tree of UDP-glucuronosyltransferases (UGTs), a major class of phase 2 enzymes, which are over-represented in genes up-regulated in *C. elegans daf-2 *mutants and long-lived dauer larvae [24]. In each case, the phylogenetic distribution of IIS-regulated genes is apparently random (Additional data file 2). Significantly, comparing worms, flies and mice, there are no orthologs for most genes in these families. In each of these large gene families, individual genes are, in most cases, the products of lineage-specific expansions [33]. This is typical of proteins whose function entails recognizing diverse chemical moieties in a changing chemical environment. Such proteins include chemoreceptors and antigen recognition proteins of the innate and acquired immune systems, as well as those involved in cellular detoxification [33,34].

### Enrichment of FOXO1-binding sites among differentially regulated genes in long-lived mutants in three species

Finally, we explored whether IIS transcriptional responses are regulated by conserved DNA binding factors. Using the program Clover (*Cis*-eLement OvEr-Representation) [35], we examined the upstream regions of the differentially expressed genes in each species for over-representation of known DNA-binding motifs (Additional data file 8). Many motifs were identified when examining each individual dataset. Of these, none was over-represented among genes regulated in the same direction in all three species. The FOXO1-binding site was over-represented among genes up-regulated in long-lived worms and mice; by contrast, this motif was over-represented among genes down-regulated in long-lived flies (Additional data file 8). Overexpression of FOXO increases lifespan in both worms and flies [27]. These findings could imply that down-regulation of FOXO-regulated genes influences lifespan in flies (perhaps lowering damage-generating processes), while up-regulation is more important in worms and mice (perhaps increasing damage-protective processes). Furthermore, an analysis using the EASE program of gene classes over-represented in genes with putative FOXO-binding sites in worms and mice revealed little similarity between these genes at this level (data not shown). Thus, while the role of FOXO in mediating transcriptional regulation by IIS shows some evolutionary conservation, the IIS-regulated target genes of FOXO may be conserved only at the level of the gene families and the biological processes that they control - not at the level of orthology.

## Discussion

### No evolutionary conservation of regulation by IIS at the level of gene orthology

The role of IIS as a regulator of aging shows evolutionary conservation. The effects of IIS on lifespan reflect the action of IIS-regulated genes and biochemistries of aging and longevity. In this study, we have asked the question: are these genes and processes public (evolutionarily conserved) or private (lineage specific)? We have done this by means of a cross-species comparison of transcript changes seen in long-lived nematodes, insects and mammals with lowered IIS when compared to normal-lived controls. To be able to do this we developed a novel, multi-level cross-species comparative method, comparing gene expression at the levels of genetic orthology, paralogy (in small and large paralog sets), and gene classes. We detected little evolutionary conservation of IIS regulation at the orthologous or paralogous gene levels. However, at the genes class or process level some evolutionary conservation was observed, including several processes previously associated with aging.

The absence of detectable regulation by IIS of orthologous genes in the three animal models tested was unexpected, for several reasons. Firstly, even if the same IIS-regulated genes did not regulate aging in worms, flies and mice, one would expect that some of the genes mediating the effects of IIS on growth and sugar metabolism would be conserved at the level of orthology. Secondly, an earlier study examined putative direct transcriptional targets of FOXO in *C. elegans *and *Drosophila*, focusing on 17 *C. elegans*-*Drosophila *ortholog gene pairs with predicted DAF-16 binding sites in their promoter regions [36]. There, a third of *C. elegans *orthologs showed IIS regulation, suggesting possible evolutionary conservation of IIS-regulated genes at the level of orthology. However, no data on IIS regulation of *Drosophila *orthologs were reported in that study. Our findings point to the opposite conclusion: that the set of genes regulated by IIS is largely lineage specific.

If significant numbers of orthologous genes were robustly IIS regulated in similar ways in multiple tissues, then it is likely that the analytical approaches that we have employed would have detected this. However, it remains possible that orthologous genes regulated similarly by IIS eluded our analysis, for several reasons. Firstly, microarray analysis may have failed to detect small but functionally significant changes in transcript levels, for example, genes showing IIS-regulated expression in only a small proportion of cells in *C. elegans *or *Drosophila*. Secondly, if the direction of IIS regulation is different in different tissues in the invertebrate models, this could prevent detection of IIS regulation. Thirdly, it may be that in extra-hepatic tissues, transcript profile changes resulting from Prop-1^df/df ^and Ghrhr^lit/lit ^are more similar to those in *C. elegans *and *Drosophila *IIS mutants. The liver consists mainly of dividing cells whereas, in the invertebrate models, adult somatic tissues consist largely of post-mitotic cells. Recent mouse studies suggest that age-related changes in gene expression may differ between mitotic and post-mitotic tissues [37]. Fourthly, gene regulation by IIS might differ between sexes (we compared data from hermaphrodite worms, females flies and male mice). Finally, although young adults of each organism were used, it is possible that the slight differences in their relative age constituted a confounding variable. More generally, the value of transcript profile studies is limited by the fact that changes in mRNA levels may not correspond to changes in levels of protein products of mRNA translation. Further studies are warranted to establish with greater certainty the extent of evolutionary conservation of regulation of genes by IIS. For example, there may be differences in the degree of evolutionary conservation of IIS regulation by direct targets of FOXO versus genes further downstream in a FOXO-regulated cascade. It would be useful to identify direct targets of FOXO, for example, using chromatin immunoprecipitation [38] and to perform cross-species comparisons of their IIS regulation.

In contrast to our studies of orthologous or paralogous genes, our comparative analysis at the gene class level identified a number of candidate gene classes and processes showing an evolutionarily conserved pattern of regulation in long-lived mutants with reduced IIS (Table 5). We performed this analysis with the aim of identifying candidate evolutionarily conserved processes that mediate the effects of IIS on aging. However, IIS is also a major regulator of growth and metabolism (including sugar homeostasis), so the presence of any of the gene categories in Table 5 may reflect a role in these other processes, rather than in aging. For example and as expected, many categories associated with sugar catabolism are up-regulated in the long-lived mutants in all three species, consistent with lowered insulin signaling. This demonstrates that methods used here are sensitive enough to identify known insulin-regulated gene categories.

Clearly, the presence of any of the gene categories in Table 5 may reflect a role in aging or in processes not linked to aging. However, a number of the gene categories present are linked to one or the other of two biological processes recently implicated in the control of aging. These are protein biosynthesis (for example, GO:0006412 protein biosynthesis, GO:0043037 translation, and GO:0045182 translation regulator activity) and GST activity (IPR004045 Glutathione-S-transferase N-terminal and IPR004046 Glutathione-S-transferase C-terminal). Data in Table 5 imply that protein biosynthesis and GST activity are down-regulated and up-regulated, respectively, in long-lived mutant worms, flies and mice. Potentially, this contributes to longevity (Figure 2).

### Decreased protein biosynthesis: a candidate longevity assurance process in multiple animal species

Several recent studies imply that increased protein biosynthesis accelerates aging. Lowered expression of a number of genes involved in mRNA translation, ribosomal proteins, translation initiation factors and ribosomal protein S6 kinase results in reduced rates of protein biosynthesis and increased lifespan in *C. elegans *[20-22]. Similarly, deletion of ribosomal protein genes can increase replicative lifespan in the budding yeast *S. cerevisiae *[19]. Over-representation of genes associated with protein biosynthesis among those down-regulated in long-lived *C. elegans*, *Drosophila *and mice implicates this process as a public, IIS-regulated mechanism controlling aging. However, it should be noted that the individual genes involved in protein biosynthesis whose expression was shown to affect *C. elegans *aging were not themselves IIS regulated [21]. How lowered protein synthesis might increase lifespan is unknown, although in *C. elegans *these perturbations increase heat stress resistance, suggesting that lowered protein synthesis leads to induction of somatic maintenance functions [21].

### GST activity: a candidate longevity assurance process in multiple animal species

GSTs detoxify a wide range of electrophilic (that is, oxidizing) and often toxic compounds by conjugation with glutathione (GSH) [39]. Such electrophiles can otherwise react with nucleophilic centers, for example, in proteins, causing molecular damage. Within biogerontology, there is a growing consensus that the primary cause of biological aging is accumulation of damage at the molecular level. Studies to date broadly support the view that longevity-assurance processes prevent accumulation of damage by promoting somatic maintenance processes [40-42]. The mechanisms involved include reduction or removal of the causes of molecular damage, and repair or turnover of damaged molecules. Thus, a role of GSTs in protection against aging is easy to rationalize.

More importantly, there is some direct experimental evidence for a role of GSTs in longevity assurance. The *C. elegans *genes *gst-5 *and *gst-10 *encode GSTs that detoxify 4-hydroxy-2-nonenal (HNE), which is a major product of peroxidation of membrane lipids and a mediator of the pathophysiological effects of oxidative stress [43]. RNAi knockdown of either of these genes reduces both HNE-conjugating activity and lifespan [23,44]. Overexpression of GST-10 or of murine mGSTA4-4 (also active against HNE) increases HNE-conjugating activity and, significantly, lifespan [23]. The over-representation of GST genes among genes up-regulated in long-lived mutant *C. elegans*, *Drosophila *and mice with reduced IIS suggests that GST activity may represent a public, IIS-regulated mechanism of longevity assurance.

The possible broader implications of the observed association between GST gene expression and extended lifespan (Table 5) may be considered in three overlapping biochemical contexts: defence against reactive oxygen species (ROS), the biology of GSH, and broad spectrum detoxification (that is, drug metabolism). GSTs play a major role in detoxifying a broad range of oxidized breakdown products of macromolecules that form during periods of oxidative stress [39]. These pro-oxidant products include α,β-unsaturated carbonyls such as HNE, hydroperoxides and epoxides. ROS such as superoxide and hydrogen peroxide have long been viewed as potential major contributors to the molecular damage that underlies aging [45]. Thus, elevated GST levels could reflect a broader up-regulation of antioxidant defenses in these three long-lived models. However, looking at transcript levels for genes encoding superoxide dismutase (SOD), which scavenges superoxide, we see that while several *sod *genes are up-regulated in *C. elegans*, this is not the case in *Drosophila *or the mouse (Table 6). Consistent with this, increased SOD has been observed in *daf-2 C. elegans *[46], but not *chico*^1^/+ *Drosophila *[8]. In terms of hydrogen peroxide scavengers, there is some evidence of increased catalase mRNA levels in long-lived *C. elegans *and *Drosophila*, but not in the mouse. In *C. elegans*, there is a tandem array of three very similar genes encoding catalase, *ctl-1*, *ctl-2 *and *ctl-3 *[47]. Our microarray analysis shows strongly increased expression of *ctl-3 *in *daf-2 *animals (*q *< 0.003); however, for the purposes of analysis in this study, *ctl-3 *data were excluded due to predicted promiscuity in probe binding between *clt-3 *and *ctl-1*. In *Drosophila *there is a possible increase in catalase mRNA levels (log2 fold change 0.3, *q *= 0.045). The absence of increased transcript levels of catalase and Mn SOD genes in Prop-1^df/df ^mouse liver was unexpected, since increased catalase levels have been reported in this tissue [48]. Overall, our transcript profile comparison provides little support for the view that direct defense against superoxide and hydrogen peroxide is a regulated public mechanism of longevity assurance.

**Table 6:** Expression of SOD genes in mutant worms, flies, mice with reduced insulin/IGF-1 signaling

	*C. elegans*			*Drosophila*			Mouse					
			
							Prop-1^df/df ^(Ames)			Ghrhr^lit/lit ^(Little)		
								
		Log2 FC	*q*		Log2 FC	*q*		Log2 FC	*q*		Log2 FC	*q*
	
Cu/Zn SOD (IC)	*sod-1*	0.66	0.073	Sod1	0.10	0.219	Sod1	0.01	0.957	Sod1	-0.12	0.616
	*sod-5*	**2.14**	**0.006**									
Cu/Zn SOD (EC)	*sod-4*	0.58	0.339	Sod3	-0.09	0.769	Sod3	0.17	0.530			
Mn SOD	*sod-2*	0.43	0.437	Sod2	0.03	0.795	Sod2	-0.03	0.903	Sod2	0.07	0.722
	*sod-3*	**4.66**	**0.000**									

Log2 FC: log2 of the fold change in mRNA transcript abundance in long-lived relative to normal-lived animals. *q*, probability that difference in mRNA abundance is the result of chance alone. Instances of significant alteration in gene expression in bold. The predicted sequence of SOD-4 suggests that it may be secreted. EC, extracellular; IC, intracellular.

A second perspective on possible GST function in aging is within the context of a broader, GSH-associated biochemistry. Besides its role in detoxification by GSTs, GSH itself acts as an antioxidant [39], and the ratio of reduced to oxidized GSH is a determinant of cellular redox status. GSH-mediated processes can clearly influence aging. For example, in *Drosophila *overexpression of glutamate cysteine ligase (γ-glutamylcysteine synthetase), the major rate-limiting enzyme in GSH biosynthesis, extends lifespan [49]. Moreover, overexpression of methionine sulfoxide reductase, an enzyme that uses GSH to restore oxidized methionine in proteins by reducing methionine sulfoxide, also increases *Drosophila *lifespan [50].

Hepatic metabolism in Prop-1^df/df ^(Ames dwarf) mice appears to be geared up for increased GSH production and usage [51-55]. Both GSH levels and GSH/GSSG ratios are increased [53], and there is increased activity of the trans-sulfuration pathway, implying increased flux of thiols from methionine to cysteine and GSH [51,55]. Possibly increased GSH production retards aging by supporting a range of mechanisms that protect against an age-related increase in levels of toxic electrophiles.

Beyond the biology of GSH, GSTs may be viewed as part of a wider system of cellular detoxification involving two phases: phase 1 (functionalization reactions), and phase 2 (conjugative reactions) [31] (Figure 3). CYPs and short-chain dehydrogenase reductases (SDRs) are major effectors of phase 1 metabolism, which through oxidative (CYP) or reductive (SDR) chemistry can bioactivate toxic molecules. Activated metabolites from phase 1 are substrates for effectors of phase 2 metabolism, such as the GSTs, UDP-glucuronosyl/UDP-glucosyltransferases (UGTs) and sulfotransferases. Phase 2 reactions can both detoxify and increase solubility of toxic moieties, aiding excretion. In mammals, this system acts in a coordinate fashion to dispose of a very broad range of xenobiotic and endobiotic compounds, including toxins, drugs, carcinogens and damaged cellular constituents [31].

Interestingly, CYPs and SDRs are also over-represented among genes up-regulated in long-lived *C. elegans*, *Drosophila *and mice (Table 5) (though UGTs and sulfotransferases are not). This suggests that the cellular detoxification more broadly might play a role in longevity assurance. Genes encoding CYPs, SDRs and UGTs are also over-represented among genes whose expression is increased in long-lived *C. elegans *dauer larvae relative to larvae that have exited the dauer stage [24,56]. In mice, caloric restriction and Prop1^df/df ^have additive effects on longevity. Phase 1 and phase 2 detoxification genes are up-regulated in both contexts and, in some cases, show additive increases in expression in Prop1^df/df ^mice subjected to caloric restriction [57]. In summary, a growing number of studies show correlations between cellular (phase 1, 2) detoxification and longevity.

Studies in *C. elegans *imply that IIS exerts its effects most strongly during the reproductive period in the first few days of adulthood [58]. This could imply that damaging aspects of protein synthesis and generation of toxins that drug-metabolizing enzymes (DMEs) protect against are elevated during this period, perhaps due to reproduction.

### An overview of evolutionary conservation of biological mechanisms controlling aging

Our results suggest that protein translation, GST activity and possibly the broader cellular detoxification system may represent 'semi-public' mechanisms of longevity determination: the processes show evolutionary conservation while the individual genes do not. In the case of GSTs, this could imply that different toxins are being cleared in different evolutionary lineages, that is, that the cause of aging, the diverse harmful molecular species that this system targets, may differ between species. Thus, although damage-causing toxins appear implicated as a cause of aging-related damage in all three species, the specific toxins involved may include some that are evolutionarily conserved and others that are lineage-specific.

The lack of gene orthology between DMEs might seem to suggest that damage-causing toxins are private. However, in at least one case this is not the case. Up-regulation of GSTs that detoxify HNE occurs both in *C. elegans daf-2 *mutants (*gst-10 *[17]) and liver of Prop1^df/df ^and Ghrhr^lit/lit ^mice (*GSTA4 *in both cases [26]), although these genes are not orthologous (Figure 1b in Additional data file 2). Moreover, expression of murine *GSTA4 *in *C. elegans *lowers HNE levels and increases lifespan [23]. This demonstrates that convergent evolution can lead to similar substrate specificities in non-orthologous DMEs. Significantly, a major source of HNE is oxidative damage to lipid, consistent with reactive oxygen species acting as a public mechanism of aging [6].

**Figure 5:**
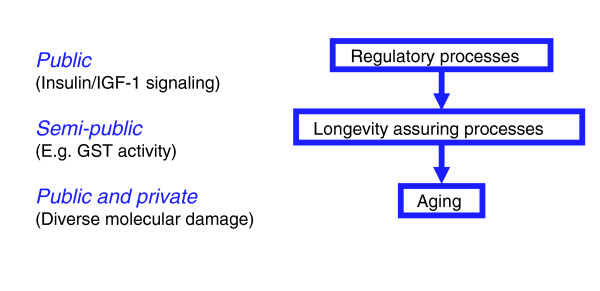
Different determinants of longevity may be public, semi-public or private. Our results suggest that public regulators of lifespan regulate semi-public mechanisms of longevity assurance, which may in turn act on a combination of private and public mechanisms of aging. The semi-public character of longevity assurance processes is reflected by the IIS-regulated gene classes. Several are linked to detoxification (such as the GSTs), and are the results of copious lineage-specific expansions.

In principle, toxins contributing to aging that are lineage-specific could contribute to the lineage specificity of aging-related pathologies. According to this view, aging involves stochastic mechanisms that are partially public and partially private. A summary overview of this interpretation is shown in Figure 5. Here, public regulators of lifespan (for example, IIS) regulate semi-public mechanisms of longevity assurance (for example, cellular detoxification), which act on both private and public types of damage generation (for example, toxins). In the specific example discussed above, IIS regulates a semi-public mechanism of longevity assurance (GSTs with HNE-conjugating activity) acting against a public mechanism of aging (HNE toxicity).

## Conclusion

We have compared changes in transcript profiles occurring in long-lived mutants with reduced IIS in *C. elegans*, *Drosophila *and the mouse. Our aim was to identify genes and processes regulated by IIS that might correspond to evolutionarily conserved (public), proximal determinants of aging. While our analysis suggests that IIS regulation of genes shows relatively little evolutionary conservation at the level of individual orthologous or paralogous genes, we identified two processes that are both IIS regulated in all three animal models, and linked to aging. In each long-lived mutant, there is evidence of lowered protein biosynthesis and increased cellular detoxification (most significantly, by GSTs). This evolutionary conservation suggests that these processes might play a role in the control of other animal lineages, for example, primates. More research is therefore needed on the impact of these two processes on aging.

## Materials and methods

### Microarray analyses

All microarray datasets analyzed in this study are publicly available. The *C. elegans *datasets are available from the Gene Expression Omnibus [59], accession number GSE1762. The *D. melanogaster *datasets are available from ArrayExpress [60], accession number E-MEXP-1099. The *M. musculus *datasets are available from ArrayExpress, accession number E-MEXP-153. For an overview of microarray datasets, see Table 1.

*D. melanogaster *used for microarray analysis were generated as follows: *chico*^1^/+ heterozygotes were selected from the progeny of a Dahomey wild type × Dahomey *chico*^1^/*CyO *cross. Wild-type Dahomey control flies were age-matched as previously described, and all flies were raised under standard culture conditions [61]. The *chico*^1 ^stock [8,62] has been maintained with continuous outcrossing to the wild-type (Dahomey) stock, where the latter was maintained in large populations to avoid inbreeding. Flies used for microarray analysis were sampled and snap-frozen in liquid nitrogen at 3 pm on day 7 of adult life (from eclosion). For each array, RNA from 20 to 30 whole flies was extracted using TRIzol (Invitrogen, Carlsbad, CA, USA) and purified with RNeasy columns (Qiagen, Valencia, CA, USA) following the manufacturer's instructions. The quality and concentration of RNA was confirmed using an Agilent Bioanalyzer 2100 (Agilent Technologies, Santa Clara, CA, USA), and further procedures followed the standard Affymetrix protocol. All samples were hybridized to the *Drosophila *Genome 2.0 Genechip. In total, five biological replicates of each genotype (wild type and *chico*^1^/+) were performed.

Information regarding the *C. elegans *and *M. musculus *(Prop1^df/df ^and Ghrhr^lit/lit^) strains, growth conditions, sample preparation and microarray hybridization protocols used to generate the raw Affymetrix data (cel files) analyzed in this study are previously described [24,26]. For our analysis of the Ames and Little mice, we used the three-month time point, which is the most similar physiologically aged time point to those used for the worm and fly microarray analyses. The sex of the animals from which mRNA was taken was as follows: *C. elegans*, hermaphrodite; *D. melanogaster*, female; *M. musculus*, male.

### Ortholog analysis

To assign gene orthologs between the three species, the Ensembl Biomart tool (Ensembl version 37) was used to download lists of orthologous genes between each species [63]. These orthologous gene-pairs generally represent the unique best reciprocal BLAST hit for the two species. For a full description of the methodology used for ortholog prediction, see the Ensembl help pages at [64]. To identify orthologous genes across all three species, we identified fly genes that had both a mouse and worm ortholog. All ortholog lists are available in Additional data file 4.

To examine the statistical significance of the number of differentially expressed orthologs that were observed when comparing the microarray datasets, we performed a simulation to determine the number of expected differentially expressed orthologs given the total population sizes (the total number of unique genes present on each microarray) and the number of differentially expressed genes in each microarray experiment. Full data from this analysis are available in Tables 2 and 3.

Distributions of common, differentially expressed orthologs were obtained by simulation, generating 10^6 ^random draws of genes. For each random draw, we drew random samples of size n_1 _and n_2 _(and n_3 _for the three species comparison) from the populations of N_1 _and N_2_, respectively (and N_3 _when necessary). Now both use subscript numerals but no italics For example, for the fly-worm up-regulation comparison, the pool of genes contained N_1 _= 10,395 fly and N_2 _= 12,414 worm genes, while the sample sizes were n_1 _= 893 and n2 = 558 up-regulated genes in fly and worm, respectively (Tables 2 and 3). The *p *value is the proportion of draws where the number of common orthologs found in the random draw was greater or equal to the number of orthologs actually observed.

### Paralog analysis

We first obtained three sets of orthology and paralogy relationships from ENSEMBL release 40, for the fly, the mouse and the worm. For each species, we created groups of in-paralogs. Genes without in-paralogs were also assigned to groups, each of which contained a single member. Orthology relationships were built initially for the three pairs of species comparisons (fly-worm, fly-mouse and worm-mouse): two groups were called orthologous when any gene from the first groups had any orthologous or paralogous relationships with any other gene from the second group (similar to single linkage clustering). For the three species comparison, three groups were called orthologous if at least one group was orthologous to the groups in both other species. A group was considered differentially expressed when at least one of its members was differentially expressed (for the analysis limited to groups of maximum size 2 or 3), or when at least half of the groups present on the chip were differentially expressed (for the analysis of all orthologous groups). The probabilities reported in additional Table 1 in Additional data file 3 were computed by drawing at random 10,000 'differentially expressed' gene lists for each (fly, worm, Ames and Little), and then computing the proportion of times that the number of common groups obtained from these random gene lists were greater or equal to the actual number of common orthologous groups.

### RNAi tests on lifespan in *C. elegans*

The potential role of conserved orthologous genes in aging was examined using an RNAi feeding protocol [65]. Bacteria expressing double-stranded RNAi for each target gene were obtained from the Ahringer RNAi feeding library [66]. One gene (Y105C5B.28) was not represented in the library, so an RNAi feeding clone was made. PCR was used to amplify a portion of Y105C5B.28 using primers JJM154 (ccagCCACCAACTACCGCC) and JJM143 (CTATCCGAACTCTAATGCTTGG). A single band of predicted size was generated, and was subcloned using the TopoTA cloning kit (Invitrogen, Carlsbad, CA, USA). This was then subcloned into the L4440 RNAi feeding vector using compatible restriction sites and transformed into the HT115(DE3) RNAi feeding bacterial strain [67]. Bacteria expressing RNAi constructs were induced overnight on NG agar plates containing 1 mM isopropyl β-D-1-thiogalactopyranoside (IPTG) as described.

For the lifespan assays, the RNAi-hypersensitive strain GA303 (*rrf-3(pk1426); daf-2(m577)*) was used to examine the effect of RNAi on the increased lifespan of *daf-2*. Eggs from gravid adult animals maintained at 20°C were isolated by hypochlorite treatment [68] and allowed to develop on RNAi plates at 20°C. L4 larvae were transferred to new plates at 25°C, and this time point was treated as day 0. Lifespan assays were then performed at 25°C as described [69], using RNAi plates throughout the experiment. The log rank test was performed using the statistical package JMP IN (SAS Institute Inc., Cary, NC, USA, version 5.1) to compare the lifespan curve of each RNAi experiment to the empty L4440 vector control. Full lifespan data are available in Additional data file 5.

### Phylogenetic analysis

Protein sequences for the genes in each of the detoxification gene families were obtained from WormBase (WS130) [70] and Ensembl (ENSEMBL 30). For genes with multiple splice forms, only one representative isoform was used for analysis, which might slightly affect the topology of the phylogenetic trees produced. For each gene family, protein alignments were computed with ClustalX using BLOSUM matrices and otherwise default settings [71,72]. During the protein alignment phase, a small number of proteins aligned dubiously with other family members, likely due to poor gene models or annotation. Such genes were removed from further analysis, or in the case of *C. elegans *were corrected by hand based on family homology [34]. Phylogenetic trees were generated from the multiple alignment using the PHYLIP package (J Felsenstein, Phylogeny Inference Package, version 3.6a2; distributed by the author, Department of Genome Sciences, University of Washington, Seattle, USA), using either protdist (Poisson-corrected distances) and neighbor-joining [73], or by *proml *using the maximum-likelihood method with one rate class. Each tree was rooted either by fungal outgroup, or center rooted. Trees were displayed and colored with Bonsai 1.2 [74]. Phylogenetic trees for each of the four detoxification gene families (CYP, GST, SDR, UGT) are available in Additional data file 2.

### Microarray statistical and computational tools

We used the following statistical and computational tools in the analysis of our microarray datasets: The R computer program (version 2.0.1) [75], Goldenspike [25], Catmap [30] and Clover [35]. For all four datasets (worm, fly, and mouse Ames and Little), raw data (cel files) were normalized, fold-changes between genotypes determined, and global statistical analysis performed, using a slightly modified version of the recently described 'Goldenspike' methodology implemented in R. Briefly, this procedure performs eight different normalization routines, which are then used to produce an average fold-change difference and false-discovery rate (*q*-value) between different genotypes that takes into consideration the variance of probe set intensity across the different normalizations. The Goldenspike methodology has been shown to out-perform most commonly used normalization methods [25]. The Goldenspike protocol was altered slightly to exclude absent probe sets (those probe sets called 'Absent' in all hybridizations by MAS5) prior to the final probe set-level Loess normalization. This alteration was found to reduce the number of false-positives associated with the absent probe sets. The output from Goldenspike for each of the datasets is available in Additional data file 9.

Prior to further analysis, we performed a quality control procedure on all three Affymetrix microarrays used in this study to ensure the specificity of each individual probe set. All individual probes have been mapped against all known and predicted transcripts of the corresponding genome using recent genome releases (*C. elegans *genome release WS140, *D. melanogaster *genome release version 4.2.1, and *M. musculus *genome release NCBIm34) [63,76,77]. This mapping allowed for up to one alignment error for either perfect match or mismatch of each individual probe, and a composite score was calculated for each probe set. This allowed each probe set to be assigned a qualitative category: perfect (all probes match a single target gene with no mismatches), promiscuous (some or all probes within a probe set map to more than one gene in the genome), weak (the probe set maps to a single gene, but some probes may have mismatches or may not map to the gene), or orphan (no probes in the probe set map to any known or predicted gene in the genome). Both promiscuous and orphan probe sets were excluded from further analysis.

To identify significant differential expression of functionally related categories of genes, we used the program Catmap [30]. We first populated this program with functional annotations for the genomes of the three species examined. To facilitate direct comparisons between the species, we used only GO and Interpro annotations, which use universal vocabularies [78,79].

For Catmap analysis, a ranked gene list based on the Bayes *t *statistic from the Goldenspike analysis was used as input. The Wilcoxon rank sum was used to generate a score based on the sum of the rankings of all genes with a particular functional annotation, and the significance of that score (the *p *value) was calculated analytically based on a random gene-rank distribution. Gene categories were considered significantly differentially regulated at a Catmap *p *value < 0.05. Full output from Catmap for each of the comparisons (up- and down-regulated genes analyzed separately) is available in Additional data file 6.

We estimated the probability of finding by chance alone *N*_obs _common gene categories among the n_1 _(or n_2_, n_3 _and n_4_) categories significantly differentially expressed in the various gene lists. To do this we performed 10,000 random draws of n_1 _(or n_2_, n_3 _and n_4 _when required) gene categories from the set of N_1 _(or N_2_, N_3_, and N_4_) gene categories annotating the genes in the first (or second, third and fourth) list. The probability is defined as the proportion of the 10,000 random draws where the number of common categories is greater or equal to N_obs_. It should be pointed out that this procedure will underestimate the true probability of finding a large number of common categories because it neglects the correlations between gene categories (see Results). A further drawback with this methodology is that drawing genes at random and performing the Catmap analysis on random ordering of genes is costly in terms of computer time and resources.

The Clover program [35] was used to identify over-representation of putative functional motifs in the 1,000 base-pairs upstream of the transcriptional start site, as defined by Ensembl [63]. Motifs in the TRANSFAC database (version 8.4) [80] were tested for statistical over-representation within the upstream region of significantly (*q *< 0.1) up- or down-regulated genes compared to the upstream sequences of all known genes. The output from Clover for each dataset is available in Additional data file 8. RepeatMasker [81] was used to mask all DNA sequences for interspersed repeats and low complexity DNA sequences.

To identify motifs that occur in the promoters of differentially expressed genes in all three species, we examined the output of Clover for motifs that were significantly over-represented (*p *value ≤ 0.05, raw score cut-off >5) in the up- or down-regulated genes in each dataset.

## Additional data files

The following additional files are available with the online version of this paper. Additional data file 1 includes legends for Additional data files 2, 3, 4, 5, 6, 7, 8, 9. Additional data file 2 is a figure showing phylogenetic trees for the four main families of drug metabolizing enzymes for *C. elegans*, *Drosophila *and mouse. Additional data file 3 includes two tables: Table 1 lists results of tests for over-representation of ortholog and paralog sets with parallel changes in gene expression; Table 2 lists the identities of genes in four paralog sets with parallel changes in gene expression in *C. elegans*, *Drosophila *and the Little mouse. Additional data file 4 contains the lists of orthologs used in this study. Additional data file 5 shows results of RNAi lifespan experiments. Additional data file 6 is the output of the Catmap analysis of the microarray data. Additional data file 7 summarizes the results of statistical tests for over-representation of gene categories identified by the Catmap analysis. Additional data file 8 contains the output of Clover analysis for gene regulatory motifs for each dataset. Additional data file 9 contains the final gene lists from our analysis or reanalysis of microarray data.

## Supplementary Material

Additional data file 1 Legends for Additional data files 2-9 Click here for file

Additional data file 2 Phylogenetic trees for the four main families of drug metabolizing enzymes for *C. elegans*, *Drosophila *and mouse Click here for file

Additional data file 3 Table 1: results of tests for over-representation of ortholog and paralog sets with parallel changes in gene expression. Table 2: identities of genes in four paralog sets with parallel changes in gene expression in *C. elegans*, *Drosophila *and the Little mouse. Click here for file

Additional data file 4 Orthologs used in this study Click here for file

Additional data file 5 Results of RNAi lifespan experiments Click here for file

Additional data file 6 Output of the Catmap analysis of the microarray data Click here for file

Additional data file 7 Summary of the results of statistical tests for over-representation of gene categories identified by the Catmap analysis Click here for file

Additional data file 8 Output of Clover analysis for gene regulatory motifs for each dataset Click here for file

Additional data file 9 Final gene lists from our analysis or reanalysis of microarray data Click here for file
