# Supplementary material for: Evolutionary conservation of regulated longevity assurance mechanisms
Source: Genome Biol. 2007 Jul 5;8(7):R132. doi: 10.1186/gb-2007-8-7-r132 (PMC2323215; doi:10.1186/gb-2007-8-7-r132)
Supplement: Additional data file 1 — Legends for Additional data files 2-9 [file gb-2007-8-7-r132-S1.doc]

**Summary of Additional, Online Materials**

**Additional Figure 1. Phylogenetic Trees of the Four Large Gene Families of Cellular Detoxification**

The phylogenetic trees of the four large gene families that are implicated in cellular detoxification: cytochrome p450’s (CYP), Short-chain dehydrogenase/reductases (SDR), Glutathione-S-transferases (GST), and UDP-glucuronosyltransferases (UGT). Differential regulation in response to reduced IIS is indicated. The methodology used to generate each tree is described in Materials and Methods. For each tree, the color of the branch indicates which species the protein derives from (*C. elegans* – green, *D. melanogaster* – blue, *M. musculus* – red). Protein names on each branch also indicate the species (ce, dm, or mm), followed by the protein name(s)/identifier(s). Branches which are faded out indicate that there was no expression data for that particular gene in our dataset, either because the gene was called ‘Absent’ in all arrays, or because the gene was not represented within our dataset. An up or down arrow next to a protein name in the tree indicates that the gene is significantly (*q* < 0.1) up or down-regulated in the long-lived mutant compared to the control in our microarray analyses.

**Additional Table 1.** The results of a statistical test for over-representation of ortholog and paralog sets with parallel regulated changes in gene expression.

**Additional Table 2.** This table shows the identity of genes in four paralog sets showing parallel expression changes in IIS mutant *C. elegans, Drosophila* and Little mice.

**Additional Dataset 1-Reciprocal Ortholog Lists**

This spreadsheet contains the list of orthologs used in our analyses. The generation of these ortholog lists is described in detail in Materials and Methods. Gene identifiers and ortholog assignments were provided by Ensembl. For each gene list, ‘orthology type’ shows the type of orthologous relationship reported in Ensembl (UBRH: unique best reciprocal hit, MBRH: Multiple best reciprocal hit), and ‘% Identity’ shows the % identity of the ortholog pair proteins between the two species, as determined during the BLASTP analysis.

**Additional Dataset 2. Effects on Lifespan of RNA-mediated Interference**

This spreadsheet contains data from the RNAi lifespan experiments. For the lifespan assays, the RNAi-hypersensitive strain GA303 (*rrf-3(pk1426); daf-2(m577)*) was used to examine the effect of RNAi on the increased lifespan of *daf-2*. Eggs from gravid adult animals maintained at 20ºC were isolated by hypochlorite treatment and allowed to develop on RNAi plates at 20ºC. L4 larvae were transferred to new plates, and this time point was used as day 0. Assays were then performed as described, using RNAi plates throughout the experiment. The Log-rank and Wilcoxon tests were performed to compare the lifespan of each RNAi experiment to the empty L4440 vector control using the statistical program JMP-in 5.1 (SAS Institute). Two replicates of each lifespan analysis were performed, and results for each experiment are shown separately and combined.

The spreadsheet contains the following information for each trial:

The trial number : Date the trial was initiated, and the researcher who performed the trial.

RNAi target – Gene targeted by RNAi

N Failed – Number of animals scored as naturally dead during the trial.

N Censored – Number of animals censored during the trial (generally due to bagging, contamination, or other non-natural death)

Mean – Mean lifespan

Std Error – Standard Error

Wilcoxon – The *p*-value calculated from the Wilcoxon test comparing the RNAi against the L4440 control for that trial.

Log-rank - The *p*-value calculated from the log-rank test comparing the RNAi against the L4440 control for that trial.

Survival curve – The survival curve for the trial.

**Additional Dataset 3. Catmap Analyses of Microarray Data**

This spreadsheet contains the output of Catmap analysis for each dataset. Each dataset was ranked according to the Bayes *t* statistic generated in Goldenspike and used as input for Catmap (see Materials and Methods). Each dataset was analyzed for both down-regulated and up-regulated categories separately (as indicated by ‘up’ or ‘down’ in the name of each sheet). For each sheet, the columns contain the following information:

Column A: Gene ontology or Interpro domain category identity

Column B: *p* value of the category as calculated by Catmap

Column C: Total number of genes in the category

**Additional Dataset 4-Statistical analysis of Catmap data**

The spreadsheet contains the results of the bootstrapping of gene categories. For each gene list, the total number of functional categories is given (all), as well as the number of categories significantly up- and down-regulated. The number of categories common between experiments is given in the Common categories table. Finally, for each comparison, the number of actual common categories is given, as well as the probability, *p*, of observing that number of common categories by chance alone, assuming independence between categories.

**Additional Dataset 5. Clover Analyses of Promoters**

This spreadsheet contains the output of the Clover analysis for each dataset (see Materials and Methods). Clover was used to separately analyze the promoters of significantly (*q* < 0.1) up-regulated and down-regulated genes in each dataset (indicated by ‘up’ or ‘down’ in the name of each sheet) to find over-represented motifs. For each sheet, the columns contain the following information:

Column A: Motif identifier from TransFac

Column B: Description of the motif from TransFac

Column C: Common name of the motif from TransFac

Column D: Consensus sequence of binding motif from TransFac

Column E: Raw score of the motif in the target set, calculated in Clover

Column F: The *p* value, indicating the probability that the presence of the motif in the target set can be explained by chance, as calculated in Clover.

**Additional Dataset 6. Final Gene Lists from Microarray Analysis**

This spreadsheet contains the final Goldenspike (GS) normalized data and gene identifiers for each microarray analysis described. The statistical analysis of each microarray experiment is described in Materials and Methods. For each sheet, the columns contain the following information:

Column A: Affymetrix probe set identifier

Column B: Mean log2 fold-change, as calculated in GS

Column C: var a, which is the pooled variance over all datasets, calculated in GS

Column D: var b, which is the variance between all the datasets, calculated in GS

Column E: The Bayes *t* statistic, calculated in GS

Column F: The false-discovery rate (*q* value), calculated in GS

Column G: The mean log2 signal for the probe set, across all datasets

Column H: The gene identifier for the probe set

Column I: When present, the common gene identifier for the probe set
