# Supplementary material for: Evolutionary conservation of regulated longevity assurance mechanisms
Source: Genome Biol. 2007 Jul 5;8(7):R132. doi: 10.1186/gb-2007-8-7-r132 (PMC2323215; doi:10.1186/gb-2007-8-7-r132)

A, Glutathione-S-transferases

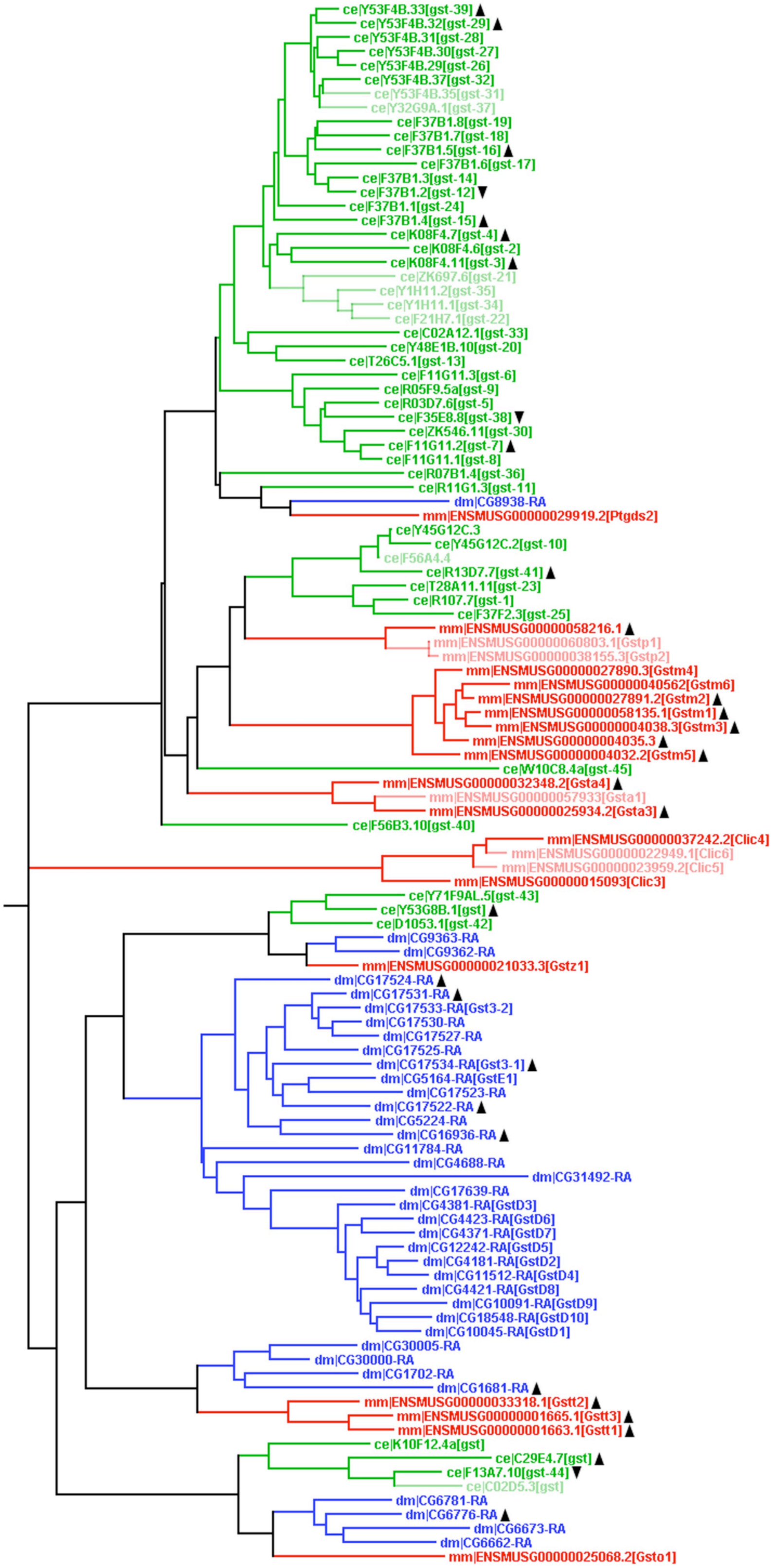

B, Cytochrome p450's

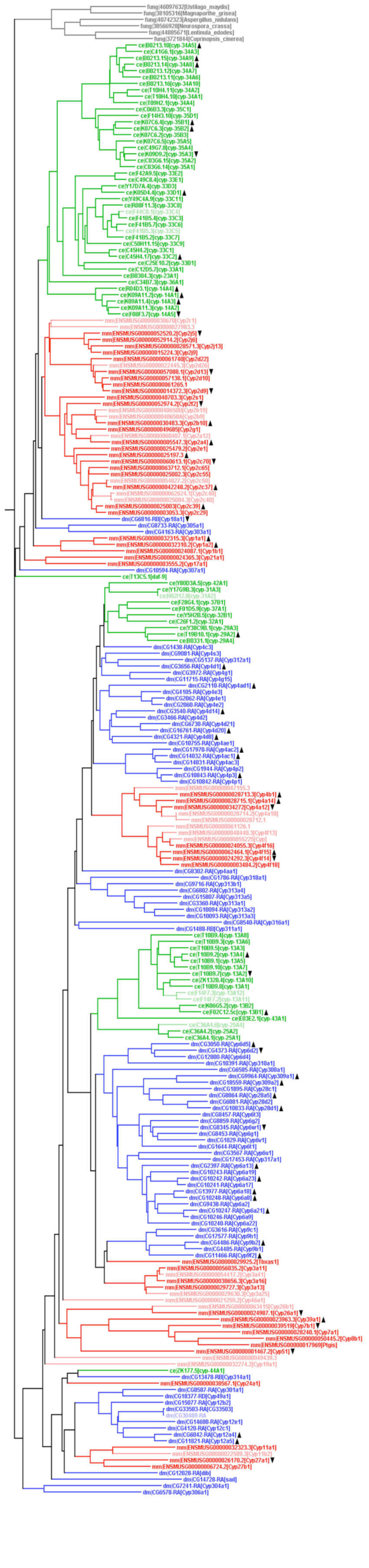

C, Short-chain dehydrogenases

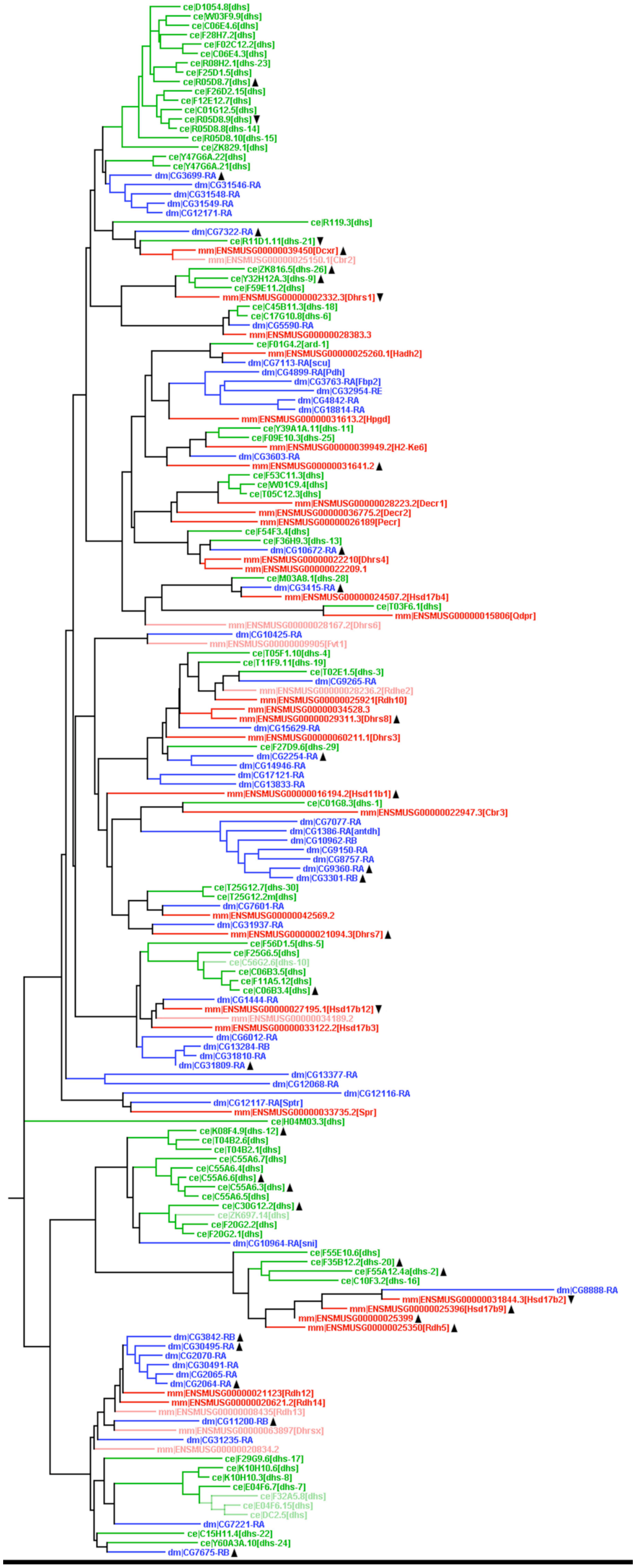

D, UDP-Glucuronosyl transferases

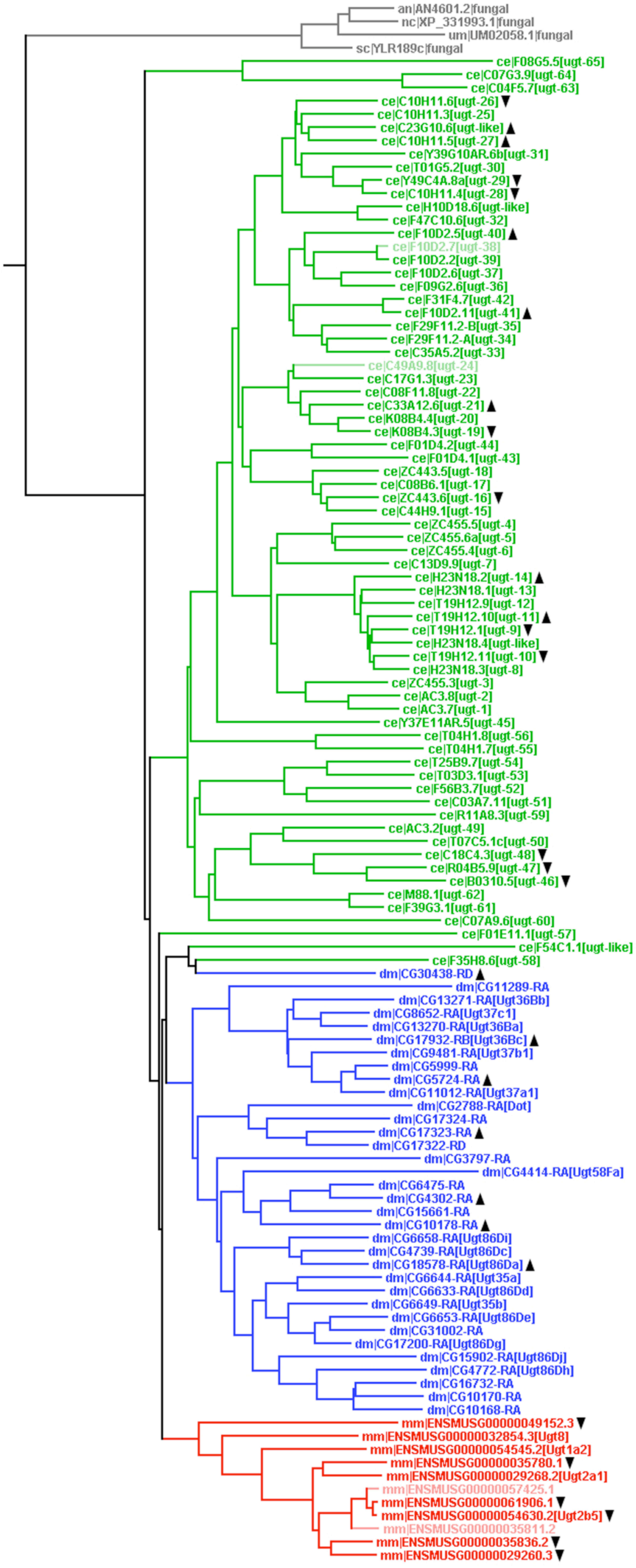

Supplement: Additional data file 2 — Phylogenetic trees for the four main families of drug metabolizing enzymes for C. elegans, Drosophila and mouse [file gb-2007-8-7-r132-S2.pdf]
