# Supplementary material for: Evolutionary conservation of regulated longevity assurance mechanisms
Source: Genome Biol. 2007 Jul 5;8(7):R132. doi: 10.1186/gb-2007-8-7-r132 (PMC2323215; doi:10.1186/gb-2007-8-7-r132)
Supplement: Additional data file 3 — Table 1: results of tests for over-representation of ortholog and paralog sets with parallel changes in gene expression. Table 2: identities of genes in four paralog sets with parallel changes in gene expression in C. elegans, Drosophila and the Little mouse. [file gb-2007-8-7-r132-S3.doc]

**Additional Table 1.** Results of statistical test for over-representation of ortholog and paralog sets with parallel regulated changes in gene expression

**Orthologs Paralog Paralog Paralog**

**sets sets <3 sets <4 sets, all**

-----------------------------------------------------------------------------------------

Shared Shared Shared Shared

Gene list* sets *p* sets *p* sets *p* sets *p*

----------------------------------------------------------------------------------------------------------

fw.up 4 0.98 9 0.94 9 0.94 10 0.90

fw.down 0 1 0 1 0 1 0 1

fa.up 8 0.92 17 0.99 17 0.99 18 0.99

fa.down 1 0.96 3 0.98 3 0.98 3 0.98

fl.up 7 0.80 25 0.31 25 0.33 27 0.22

fl.down 0 1 1 0.99 1 0.99 1 0.99

wa.up 5 0.60 15 0.30 16 0.24 18 0.11

wa.down 3 0.63 6 0.80 7 0.68 7 0.70

wl.up 4 0.57 12 0.27 13 0.19 14 0.13

wl.down 0 1 1 0.99 2 0.97 2 0.97

fwa.up 0 1 1 0.69 1 0.70 1 0.703

fwa.down 0 1 0 1 0 1 0 1

fwl.up 0 1 4 **0.013** 4 **0.013** 4 **0.013**

fwl.down 0 1 0 1 0 1 0 1

fwal.up 0 1 0 1 0 1 0 1

fwal.down 0 1 0 1 0 1 0 1

----------------------------------------------------------------------------------------------------------

Ortholog sets: the number of sets (pairs, triplets, quadruplets) of predicted orthologous genes showing significant changes in transcript levels in the named combination of species, and direction. Paralog sets: as for ortholog sets, but here data is shown for sets of paralogs (both orthologs and non-orthologous homologs) of varying sizes, either <3 (i.e. one or two genes per species), <4 (1-3 genes per species), or of any size. In the case of paralog sets of size <3 or <4, the number of sets with at least 1 differentially expressed gene per species is shown. In the case of paralog sets of all sizes, sets where at least 50% of genes showed differential expression are shown. This 50% cut off excludes some gene classes identified in the Catmap analysis, e.g. glutathione-S-transferases.

*p* is the probability of this number of shared sets being seen by chance alone (shown in bold where *p* < 0.05).

*f, fly; w, worm; a, Ames mouse; l, Little mouse. fl.up, up-regulated in both long-lived fly and worm models. fl.down, down-regulated in both long-lived fly and worm models; and so on.

**Additional Table 2.** Identity of genes in four paralog sets showing parallel expression changes in IIS mutant *C. elegans, Drosophila* and Little mice.

**Mouse**

**Worm Fly Little Ames**

**gene log2 FC *q* gene log2 FC *q* gene* log2 FC *q* gene* log2 FC *q***

--------------------------------------------------------------------------------------------------------------------------------------------------------------------------

**Paralog set 1: Fructose-biphosphate aldolase**

**F01F1.12 0.14 0.046** CG5432 017390 -0.46 0.029 017390 0.032 0.88

T05D4.1 -0.037 0.64 **CG6058 0.485 0.018** **028307 0.33 0.096** 028307 0.28 0.11

030695 030695

059343 059343

063129 063129

067382 067382

**Paralog set 2: Pantothenate kinase**

**C10G11.5 0.26 0.0012** **CG5725 0.253 0.092** 018846 018846

C42D8.3 0.088 0.21 CG5828 -0.0374 0.75 029056 029056

**033610 0.86 0.0014** 033610 -0.012 0.95

037514 037514

**Paralog set 3: β-glucosidase, lactase phlorizinhydrolase, and related proteins**

**C50F7.10 0.18 0.013** **CG9701 0.287 0.072** 026354 026354

E02H9.5 -0.15 0.031 **029195 0.52 0.015** 029195 0.27 0.12

032401 032401

058488 058488

067313 067313

**Paralog set 4: Glycerol-3-phosphate dehydrogenase**

**T25G3.4 0.12 0.079** CG2137 -0.013 0.91 **026827 0.75 0.0030** 026827 -0.88 0.00035

Y50E8A.6 **CG8256 0.33 0.052**

--------------------------------------------------------------------------------------------------------------------------------------------------------------------------

Log2 FC: log2 fold change in mRNA transcript abundance in long-lived compared to normal-lived animals. Thus, where the log2 FC is negative, the gene is down-regulated in the long-lived form. *q*, false discovery rate (probability of such a change in transcript abundance occurring by chance alone). In bold, genes that are significantly up-regulated.

*For all mouse gene names, the prefix ENSMUSG00000 to give the full name.

Where no data is given for a given gene, this is usually because no significant hybridization to the probe set was detected.
